# Patients with knee osteoarthritis demonstrate improved gait pattern and reduced pain following a non-invasive biomechanical therapy: a prospective multi-centre study on Singaporean population

**DOI:** 10.1186/1749-799X-9-1

**Published:** 2014-01-02

**Authors:** Avi Elbaz, Amit Mor, Ganit Segal, Yoav Aloni, Yee Hong Teo, Yee Sze Teo, Shamal Das-De, Seng Jin Yeo

**Affiliations:** 1 AposTherapy Research Group, Herzliya, Israel; 2 AposTherapy Research Group, Singapore, Singapore; 3 Department of Orthopaedic Surgery, Tan Tock Seng Hospital, Singapore, Singapore; 4 Department of Orthopaedic Surgery, Changi General Hospital, Singapore, Singapore; 5 Department of Orthopaedic Surgery, Yong Loo Lin School of Medicine, National University of Singapore, Singapore, Singapore; 6 Division of Foot and Ankle Surgery, National University Health System, Singapore, Singapore; 7 Department of Orthopaedic Surgery, Singapore General Hospital, Singapore, Singapore

**Keywords:** Knee, Osteoarthritis, Gait, Pain, Biomechanical device

## Abstract

**Background:**

Previous studies have shown the effect of a unique therapy with a non-invasive biomechanical foot-worn device (AposTherapy) on Caucasian western population suffering from knee osteoarthritis. The purpose of the current study was to evaluate the effect of this therapy on the level of symptoms and gait patterns in a multi-ethnic Singaporean population suffering from knee osteoarthritis.

**Methods:**

Fifty-eight patients with bilateral medial compartment knee osteoarthritis participated in the study. All patients underwent a computerized gait test and completed two self-assessment questionnaires (WOMAC and SF-36). The biomechanical device was calibrated to each patient, and therapy commenced. Changes in gait patterns and self-assessment questionnaires were reassessed after 3 and 6 months of therapy.

**Results:**

A significant improvement was seen in all of the gait parameters following 6 months of therapy. Specifically, gait velocity increased by 15.9%, step length increased by 10.3%, stance phase decreased by 5.9% and single limb support phase increased by 2.7%. In addition, pain, stiffness and functional limitation significantly decreased by 68.3%, 66.7% and 75.6%, respectively. SF-36 physical score and mental score also increased significantly following 6 months of therapy (46.1% and 22.4%, respectively) (*P* < 0.05 for all parameters).

**Conclusions:**

Singaporean population with medial compartment knee osteoarthritis demonstrated improved gait patterns, reported alleviation in symptoms and improved function and quality of life following 6 months of therapy with a unique biomechanical device.

**Trial registration:**

Registration number NCT01562652.

## Background

Osteoarthritis (OA) is the most prevalent form of arthritis [1]. About 6% of Asian males and 12% of Asian females suffer from knee OA [2]. The prevalence of OA increases with age and generally affects women more frequently than men. The population of many Asian countries are ageing rapidly, and it is estimated that between 2008 and 2040, the proportion of the Singaporean population aged 65 years old and over will increase by 316% [3]. Hence, the prevalence of knee OA is expected to rise.

Knee OA is associated with symptoms of pain, functional disability and deteriorated quality of life that might lead to further morbidity; 10% of Asian males and 13% of Asian females report knee pain [2]. From a social perspective, OA is costly, having high direct costs in the form of increased utilization of hospital and medical services and also high indirect costs through lost productivity of individuals [4,5]. Therefore, researchers are constantly trying to find effective treatments that will help to halt the disease progression and even reverse it.

Patients with knee OA demonstrate pathological gait patterns compared to age-matched controls [6,7]. Specifically, patients with knee OA demonstrate a deterioration in spatio-temporal gait parameters including slower walking velocity, shorter step length and shorter single-limb support (SLS) compared to matched controls [6,8]. Recent studies have reported an association between the level of symptoms (i.e. pain and functional limitation) of knee OA patients and their gait pattern [9]. Elbaz et al. have published a new objective functional classification of patients with knee OA which is based on the patient’s ability to bear single loads on one knee while the contralateral leg swings forward (i.e. single limb support) [10]. This new classification is thought to give a clearer description of the patient’s functional condition than radiographic findings, considering the knowledge that the correlation between symptoms and radiographic changes is poor [9,11].

Several non-invasive interventions exist for knee OA; amongst them are biomechanical interventions. Previous studies have shown the effect of several biomechanical interventions focused on foot centre of pressure manipulation and agility and perturbation training in patients with knee OA [12-17]. A relatively new biomechanical intervention for patients with knee OA was introduced. This intervention incorporates a personalized foot-worn biomechanical device and treatment methodology for patients with knee OA (AposTherapy). Recent studies have found that patients with knee OA who underwent this intervention reported significant improvements in the levels of pain and function [12,13]. Furthermore, improvements were also found in the gait patterns of these patients, muscle activation patterns [18] and knee adduction moment, which is highly associated with disease severity [14]. All of the abovementioned articles were conducted on a Caucasian western population, and information regarding the effect of this therapy on the Asian population is missing. The purpose of this study was to evaluate the effect of this special intervention on the level of symptoms and gait patterns in a multi-ethnic Singaporean population suffering from knee OA.

## Methods

### Participants

**Figure 1:**
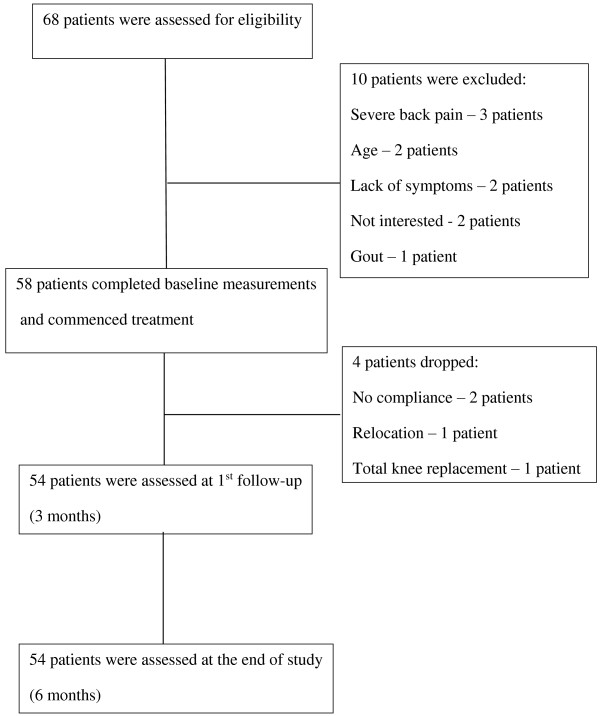
Flow chart of assessment, enrolment and follow-up.

Sixty-eight patients were assessed at baseline. Ten patients were excluded as they did not meet the inclusion criteria. Overall, 58 patients (39 females and 19 males) diagnosed with primary medial compartment knee OA participated in this study, and 54 patients completed it (Figure 1). Ninety-five percent of the patients (49 patients) had bilateral knee OA. The mean (standard deviation (SD)) age was 59.7 (6.1) years and mean (SD) body mass index (BMI) was 30.7 (14.6) kg/m^2^. Forty-four patients (82%) were Chinese, five patients (9%) were Indian and five patients (9%) were Malay. Patients’ structural OA severity was determined by the Kellgren and Lawrence (KL) score [19]. Twenty patients (37.0%) were graded 2, 21 patients (38.9%) were graded 3 and 13 patients (24.1%) were graded 4.

Patients were referred to the therapy centre by their physician after being diagnosed with primary medial compartment knee OA. The study protocol was approved by Parkway Independent Ethic Committee and is registered in clinicaltrial.gov website (registration number NCT01562652). All patients signed their informed consent after understanding the study’s objectives and protocol. Inclusion criteria were (1) patients suffering from symptomatic bilateral knee OA at the medial compartment for at least 6 months, fulfilling the American College of Rheumatology clinical criteria for OA of the knee [20], and having radiographically assessed OA of the knee classified according to Kellgren and Lawrence score [19]; (2) males and females above 50 years old.

Exclusion criteria were (1) patients suffering from acute septic arthritis, (2) patients suffering from inflammatory arthritis, (3) patients who received a corticosteroid injection within 3 months of the study, (4) patients suffering from avascular necrosis of the knee, (5) patients with a history of knee buckling or recent knee injury, (6) patients who have had a joint replacement, (7) patients suffering from neuropathic arthropathy, (8) patients with an increased tendency to fall (more than three falls in the last year), (9) patients with a history of pathological osteoporotic fracture and (10) patients suffering from severe symptomatic degenerative arthritis in lower limb joints other than the knees.

### Gait analysis

A computerized mat was used to measure spatio-temporal gait parameters (GaitMat™ II system, E.Q., Inc. Chalfont, PA, USA). The validity and reliability of the electronic gait mat was reported previously [21]. During the gait test, all patients walked barefoot at a self-selected speed. Patients walked 3 m before and after the walkway mat to allow sufficient acceleration and deceleration time outside the measurement area. Each gait test included six walks, and the mean value of the six walks was calculated for each parameter. The following spatio-temporal parameters were evaluated: velocity (cm/s), step length (cm), stance phase (% gait cycle) and SLS phase (% gait cycle).

### Self-reported questionnaires

To examine changes in pain, function and quality of life perception, the translated Singaporean version of the Western Ontario and McMaster Universities Arthritis Index (WOMAC) questionnaire [22,23] and SF-36 Health Survey [24,25] were evaluated. The WOMAC questionnaire is a visual analogue scale (VAS) ranging from 0 to 10 cm, with 0 cm indicating no pain or limitation in function and 10 cm indicating the most severe pain or limitation in function. The questionnaire contains 24 questions of which 5 evaluate pain, 2 evaluate joint stiffness and 17 evaluate function. The SF-36 is scored between 0 and 100, with 0 indicating the worst quality of life and 100 indicating the best quality of life. The questionnaire contains 36 questions of which two subscales are calculated to give a physical and mental score. The physical score is composed of questions regarding physical health, role limitation due to physical problems, pain, general health and vitality. The mental score is composed of questions in the fields of general health, vitality, social functioning, role limitation due to emotional problems and emotional well-being.

### Intervention

**Figure 2:**
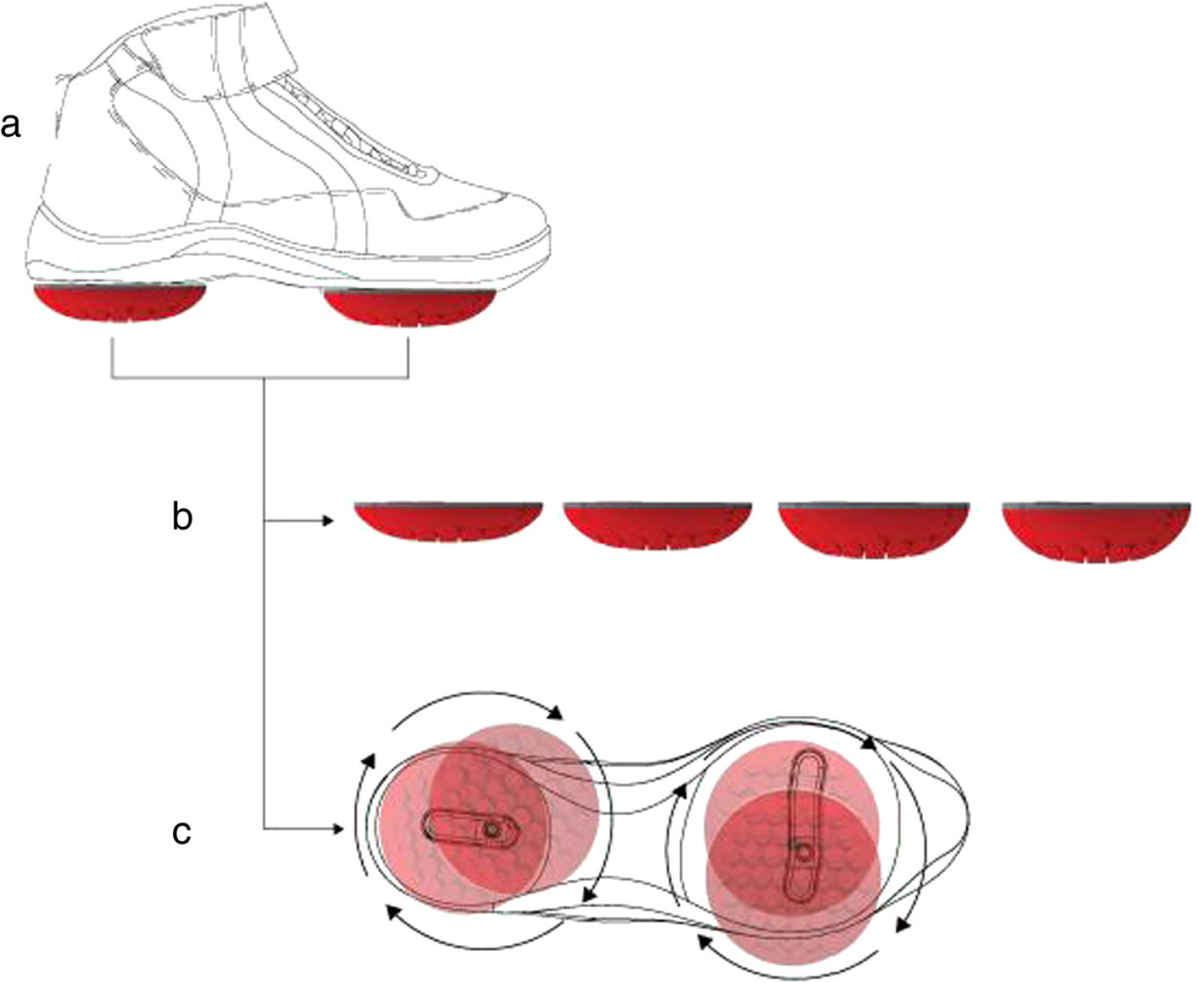
**Apos System. (*a*)** Biomechanical device comprising two individually calibrated elements and a foot-worn platform. The elements are attached to a platform under the hindfoot and forefoot regions. **(*b*)** The biomechanical elements are available in different degrees of convexity and resilience. **(*c*)** The specially designed sole of the platform includes two mounting rails and a positioning matrix to enable flexible positioning of each biomechanical element.

A novel foot-worn biomechanical device (Apos System, APOS—Medical and Sports Technologies Ltd. Herzliya, Israel) comprising convex adjustable pods placed under the hindfoot and forefoot regions of each foot was used. This device enables customized calibration of the pods (i.e. biomechanical elements) which allows manipulation of the centre of pressure passing through the knee joint, hence enabling control of the external forces (i.e. coronal and sagittal moments) acting on the knee joint [26-28]. Furthermore, the convexity of the biomechanical elements promotes minor perturbation throughout all phases of the step cycle (Figure 2) and trains neuromuscular control [29].

### Study protocol

Prior to each assessment, patients were instructed not to consume pain medication for at least 72 h in order to eliminate the effect of these medications on the patient’s pain levels and gait patterns. Anthropometric measurements were taken at baseline. All patients underwent a gait test on the computerized mat and completed the WOMAC questionnaires and the SF-36 Health Survey during each visit to the therapy centre. After the completion of the baseline measurements, the biomechanical device was individually calibrated to each patient by a physiotherapist certified in the AposTherapy methodology. Patients received exercise guidelines from the therapist. They were instructed to put on the device and go about their daily activities for 10 min once a day (accumulating 5-min walks) during the first week and gradually increasing to 60 min once a day (accumulating between 25- and 30-min walks) for the rest of the treatment period. Patients were re-evaluated after 3 and 6 months of treatment. Patients were instructed not to consume any other treatment modalities during the study period including physical therapy, injections and NSAIDs.

### Statistical analysis

All spatio-temporal gait parameters and self-evaluation questionnaire scores were presented as mean (SD), followed by 95% confidence interval for all time periods. Non-parametric one-sample Kolmogorov-Smirnov tests were calculated to compare the observed cumulative distribution function for the continuous variables with the normal theoretical distribution. The general linear model repeated measures procedure was used to provide analysis of variance for gait parameters and self-evaluation questionnaires when the same measurement was made three times on each subject. Repeated measures ANOVA were conducted by subgroups to demonstrate the sensitivity of the results. Data were analyzed with IBM SPSS software version 19.0, and the significance level was set at 0.05.

## Results

Fifty-four patients out of fifty-eight recruited patients completed the study (93.1%). Four patients did not complete the study: two patients did not comply with the treatment, one patient relocated and could not continue with therapy and one patient chose to undergo a total knee replacement. All remaining patients complied with the treatment, and there were no reports of any adverse events during the treatment period.

All spatio-temporal gait parameters significantly improved following 3 months of therapy except for SLS phase of the less symptomatic knee. After 6 months of therapy, all parameters improved significantly compared to baseline. Specifically, gait velocity improved by 15.9%, step length of the more symptomatic knee improved by 10.3%, the stance phase of the more symptomatic knee decreased by 5.9% and the SLS phase of the more symptomatic knee increased by 2.7%. The changes in gait parameters throughout the treatment are summarized in Table 1.

**Table 1:** Changes in spatio-temporal gait parameters following 6 months of therapy

**Parameter**	**Baseline**	**3 months**	**6 months**	***P *** **value**
Velocity (cm/s)	86.9 (16.4)	98.8 (16.1)	100.7 (16.5)	<0.001
	[82.4–91.3]	[94.4–103.2]	[96.2–105.2]	
Step length MS (cm)	49.4 (7.9)	53.7 (7.3)	54.5 (7.5)	<0.001
	[47.3–51.6]	[51.7–55.7]	[52.5–56.6]	
Step length LS (cm)	50.0 (7.5)	54.0 (7.2)	54.7 (7.8)	<0.001
	[48.0–52.1]	[52.0–56.0]	[52.6–56.8]	
Stance MS (% GC)	71.4 (7.3)	67.3 (6.9)	67.2 (7.4)	<0.001
	[69.4–73.3]	[65.4–69.2]	[65.2–69.2]	
Stance LS (% GC)	72.8 (7.4)	67.2 (8.1)	67.4 (6.5)	<0.001
	[70.7–74.8]	[65.0–69.5]	[65.6–69.2]	
Single-limb support MS (% GC)	37.2 (2.6)	37.2 (2.5)	38.1 (2.5)	0.001
	[36.5–37.9]	[37.5–38.8]	[37.4–38.8]	
Single-limb support LS (% GC)	38.7 (2.3)	39.0 (1.9)	39.2 (1.9)	0.027
	[38.1–39.4]	[38.5–39.5]	[38.7–39.7]	

Results are presented as mean (SD) [95% CI]. *MS* more symptomatic, *LS* less symptomatic, *GC* gait cycle. *S*et *P* < 0.05.

**Figure 3:**
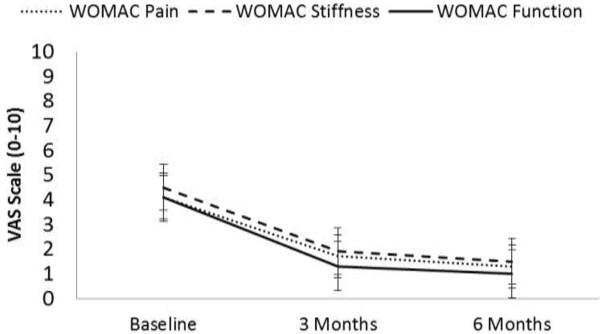
**Changes in WOMAC pain, stiffness and function following 6 months of therapy.** The WOMAC questionnaire includes 24 questions in a VAS format (0 = no pain/stiffness/difficulty, 10 = severe pain/stiffness/difficulty).

The results of the self-evaluation questionnaires improved significantly over time. The following are the results of all subcategories of the WOMAC questionnaires: WOMAC pain decreased by 68.3%, WOMAC stiffness decreased by 66.7% and WOMAC functional limitation decreased by 75.6% following 6 months of therapy (*P* < 0.001 for all). Figure 3 illustrates the changes over time in the three WOMAC subcategories.

All of the SF-36 subscales improved significantly following 3 months of therapy except for emotional well-being. The SF-36 physical score (average of five physical subscales) and the SF-36 mental score (average of five mental subscales) increased significantly over time. After 6 months of therapy, all subscales improved significantly except for emotional well-being. Summarized results are presented in Table 2.

**Table 2:** Changes in SF-36 quality of life following 6 months of therapy

**Parameter**	**Baseline**	**3 months**	**6 months**	***P *** **value**
Physical function	45.2 (20.1)	56.9 (19.7)	61.9 (21.5)	<0.001
	[39.7–50.7]	[51.6–62.3]	[56.0–67.7]	
Limitation due to physical health	29.8 (34.0)	55.3 (42.4)	75.0 (33.6)	<0.001
	[20.4–39.3]	[43.5–67.1]	[65.7–84.3]	
Limitations due to emotional problems	59.8 (44.5)	71.8 (39.8)	84.6 (28.4)	<0.001
	[47.4–72.2]	[60.7–82.9]	[76.7–92.5]	
Vitality	52.5 (17.2)	58.9 (17.0)	60.3 (17.8)	0.004
	[47.8–57.2]	[54.2–63.5]	[55.4–65.1]	
Emotional well being	68.0 (10.9)	69.9 (11.7)	71.0 (9.5)	0.061
	[65.1–71.0]	[66.7–73.0]	[68.5–73.6]	
Social functioning	65.6 (22.9)	73.8 (21.9)	75.7 (19.9)	0.004
	[59.3–71.8]	[67.9–79.8]	[70.3–81.2]	
Pain	48.3 (20.0)	63.4 (20.7)	62.3 (21.3)	0.001
	[42.8–53.8]	[57.7–69.0]	[56.4–68.1]	
General health	50.5 (19.1)	60.1 (19.1)	66.3 (17.1)	<0.001
	[44.3–55.7]	[54.9–65.3]	[61.6–70.9]	
Physical Score	44.7 (14.5)	59.0 (18.0)	65.3 (17.7)	<0.001
	[40.8–48.7]	[54.1–64.0]	[60.5–70.1]	
Mental Score	58.5 (16.0)	67.0 (16.3)	71.7 (13.4)	<0.001
	[54.2–62.9]	[62.5–71.4]	[68.0–75.3]	

SF-36 Health Survey includes 36 questions. Results range between 0 and 100 (0 = poor quality of life, 100 = high quality of life). Set *P* < 0.05.

A further analysis was conducted on the SLS phase changes. We evaluated the changes in SLS based on Elbaz et al.’s functional severity classification of knee OA [10]. According to this classification, SLS is divided into five groups, each indicating a different functional severity level. Patients who fall into Q1 are characterized with poor walking abilities and high levels of pain and functional limitation, and patients who fall into Q5 are characterized with normal walking abilities and low levels of pain and functional limitation. At baseline, 11.1% of the patients fell into Q1, 7.5% fell into Q2, 25.9% fell into Q3, 22.2% fell into Q4 and 33.3% of the patients fell into Q5. After 6 months of therapy, a considerable shift was noticed: 5.5% of the patient fell into Q1, 5.5% fell into Q2, 11.1% fell into Q3, 25.9% fell into Q4 and 52.0% of the patients fell into Q5.

We also examined the effect of BMI and KL grading on the level of improvement following treatment. A sensitivity analysis divided the cohort into two groups based on their BMI values (below/above 26.4 which represents the median). A second analysis divided the cohort into two groups based on their KL grade (KL 1–2 and KL 3–4). We examined the changes in the measured variables in each subgroup. In the BMI subgroup analysis, most of the variables maintained the trend and improved significantly, except for SLS of the less symptomatic leg in both BMI subgroups. Furthermore, the following SF-36 subcategories of the heavier BMI group became non-significant: vitality, social functioning and pain. In the KL grade subgroup analysis, most of the variables maintained the trend and improved significantly, except for the SLS of the more and less symptomatic leg and the vitality subcategory of the SF-36 in the more severe KL group. Furthermore, the social functioning subscale of SF-36 in the less severe KL group also became non-significant.

## Discussion

The purpose of the present study was to evaluate the effect of a non-invasive foot-worn biomechanical device on the spatio-temporal gait patterns, level of pain, function and quality of life of Singaporean patients suffering from medial compartment knee OA. Following 3 months of therapy, patients demonstrated an improvement in gait patterns and alleviation in symptoms. Patients maintained these improvements after 6 months of therapy. The improvement in gait pattern was accompanied by a reduction in pain and joint stiffness and with an increase in function and quality of life. All of the SF-36 quality of life subcategories improved significantly except for emotional well-being (*P* = 0.06). A possible explanation might be the relatively high pre-treatment value of the study population. These results support previous prospective studies that examined this therapy on a western Caucasian population. Bar-Ziv et al. reported a 65% reduction in pain and a 63% reduction in functional disability following 2 months of AposTherapy [13]. Haim et al. reported a 61% reduction in pain and a 63% reduction in functional disability following 9 months of AposTherapy [14]. Patients in the current study reported a mean reduction of 68% in pain and a mean reduction of 76% in functional disability following 6 months of therapy. In addition, the reduction in pain and functional disability and the improvement in quality of life in the current study met with the OMERACT-OARSI criteria and that of Angst et al. for the amount of improvement needed to assure clinical significance to the patient [30,31]. Based on this, it may be assumed that the examined therapy in the current study might have a positive effect on the level of pain and functional performance of the Singaporean population suffering from knee OA.

Currently, the management of knee OA focuses on alleviating the patient’s symptoms, which is usually monitored using self-reported questionnaires. Since questionnaires are subjective, there is a need for an objective evaluation tool to help assess symptoms. Recently, it has been suggested that computerized gait analysis might serve as a good indicator for functional severity. Elbaz et al. presented an objective functional severity classification which is based on the patient’s ability to bear loads on the painful knee (SLS) [10]. It is assumed that the ability of a patient to bear loads on the painful knee will decrease as pain level increases, which will also compromise the patient’s functional abilities. In the current study, we examined the changes in SLS following therapy based on the abovementioned classification. At baseline, only 35% of the patients were placed at the fifth quintile, which corresponds with low levels of pain and functional disability. After 6 months of therapy, 52% of the patients were placed at the fifth quintile. These results objectively support the patient’s perception and strengthen the notion of reduced pain and improved function.

The presentation of spatio-temporal gait parameters can be either in absolute values (s) or as percentage of gait cycle time (% GC) [32]. A reduction in the absolute stance phase time is considered to reflect an antalgic gait. However, an increase in the relative stance phase represents an abnormal gait pattern that usually characterizes knee OA in patients [33]. It is important to stress that in the current study, patient’s had above-normal stance phase values (% GC) before commencing treatment. Following treatment, a reduction in stance was noted. The reduction in stance phase was accompanied with an increase in gait velocity bringing the patient closer to normal values and maintaining symmetry [33,34]. We therefore considered the reduction in stance phase a positive outcome.

We examined the effect of BMI and KL grade on the examined gait parameters and questionnaires. BMI and KL grade had a minor effect on the results following a sensitivity analysis and did not affect the significant improvement in pain and function. In the BMI sensitivity analysis, *P* values were close to significant; therefore, we believe that this trend was not due to BMI difference between patients. Similar to the BMI sensitivity analysis, the results in the KL sensitivity analysis are also probably due to a small sample size and not directly because of the KL grade. This corresponds with other publication that reported poor correlation between knee symptoms and radiographic grading [9]. In order to better understand the effect of BMI and KL grading on the outcomes of this treatment, we recommend that a larger cohort should be examined.

This study has some limitations. Firstly, the study lacked a control group. A previous study, however, by Bar-Ziv et al. [13] had already demonstrated the positive effect of this therapy compared to a control group in a double-blind study. Secondly, this study did not include radiographic assessment of the patients’ knees. Radiographic evaluation of structural changes in the knee joint is an integral process in knee OA assessment. The correlation, however, between structural severity and knee OA symptoms is poor [9]. Thirdly, the study examined patients with medial compartment knee OA; hence, the results are applicable for this type of knee OA. Furthermore, this study included patients with severe degenerative changes to the knee joint which may bias the results as other compartments of the knee are also involved. Future studies should examine the effect of this therapy on structural changes at the knee joint and should also examine the effect of this therapy in patients with lateral/anterior knee OA and in patients with different knee alignment (varus/valgus). Lastly, follow-up period was relatively short. A recent long-term follow-up study by Bar-Ziv et al. demonstrated maintenance of improved symptoms in patients with medial knee OA [35]; however, this should be applied on the Singaporean population and include gait assessment.

## Conclusions

Singaporean population with medial compartment knee OA demonstrated improved gait patterns, reported alleviation in symptoms and improved function and quality of life following 6 months of AposTherapy. The results of this study support previous works that examined the effect of this therapy on western population. Based on the results of this study, it may be assumed that this therapy might have a positive effect on patients with knee OA and should be considered as an additional treatment modality in the management of knee OA.

## Competing interests

Avi Elbaz and Amit Mor hold shares in Apos Medical and Sports Technologies Ltd (‘Apos’). Ganit Segal and Yoav Aloni are salaried employees of Apos. All other authors are co-researchers in a number of studies. They do not receive and are not entitled to any financial compensation from Apos.

## Authors’ contributions

AE and AM have made substantial contributions to the conception, design and interpretation of data. GS has made substantial contributions to the conception, design, analysis and interpretation of data. She has also been involved in drafting the manuscript. YA, HTY, STY, SDD and JYS have made substantial contributions to the conception, design and acquisition of data. All authors have been involved in revising the manuscript critically for important intellectual content and have read and approved the final manuscript.
